# Design and methodology of the Swiss Transplant Cohort Study (STCS): a comprehensive prospective nationwide long-term follow-up cohort

**DOI:** 10.1007/s10654-012-9754-y

**Published:** 2013-04-02

**Authors:** Michael T. Koller, Christian van Delden, Nicolas J. Müller, Philippe Baumann, Christian Lovis, Hans-Peter Marti, Thomas Fehr, Isabelle Binet, Sabina De Geest, Heiner C. Bucher, Pascal Meylan, Manuel Pascual, Jürg Steiger

**Affiliations:** 1 Basel Institute for Clinical Epidemiology and Biostatistics, University Hospital Basel, Basel, Switzerland; 2 Service of Transplantation, Department of Surgery, University Hospitals Geneva, University of Geneva, Geneva, Switzerland; 3 Division of Infectious Diseases and Hospital Epidemiology, University Hospital Zurich, Zurich, Switzerland; 4 Division of Medical Information Sciences, University Hospitals of Geneva, University of Geneva, Geneva, Switzerland; 5 Department of Nephrology and Hypertension, Inselspital, University of Bern, Bern, Switzerland; 6 Division of Nephrology, University Hospital Zurich, Zurich, Switzerland; 7 Nephrology/Transplantation Medicine, Kantonsspital, St. Gallen, Switzerland; 8 Institute of Nursing Science, University of Basel, Basel, Switzerland; 9 Institute of Microbiology and Infectious Diseases Service, University Hospital Center and University of Lausanne, Lausanne, Switzerland; 10 Transplantation Centre, University Hospital Center and University of Lausanne, Lausanne, Switzerland; 11 Transplantation Immunology and Nephrology, University Hospital Basel, Basel, Switzerland

**Keywords:** Transplantation, Cohort, Design, Outcome research

## Abstract

In Switzerland, organ procurement is well organized at the national-level but transplant outcomes have not been systematically monitored so far. Therefore, a novel project, the Swiss Transplant Cohort Study (STCS), was established. The STCS is a prospective multicentre study, designed as a dynamic cohort, which enrolls all solid organ recipients at the national level. The features of the STCS are a flexible patient-case system that allows capturing all transplant scenarios and collection of patient-specific and allograft-specific data. Beyond comprehensive clinical data, specific focus is directed at psychosocial and behavioral factors, infectious disease development, and bio-banking. Between May 2008 and end of 2011, the six Swiss transplant centers recruited 1,677 patients involving 1,721 transplantations, and a total of 1,800 organs implanted in 15 different transplantation scenarios. 10 % of all patients underwent re-transplantation and 3% had a second transplantation, either in the past or during follow-up. 34% of all kidney allografts originated from living donation. Until the end of 2011 we observed 4,385 infection episodes in our patient population. The STCS showed operative capabilities to collect high-quality data and to adequately reflect the complexity of the post-transplantation process. The STCS represents a promising novel project for comparative effectiveness research in transplantation medicine.

**Electronic supplementary material:**

The online version of this article (doi:10.1007/s10654-012-9754-y) contains supplementary material, which is available to authorized users.

## Introduction

In Switzerland, solid organ donor evaluation and organ allocation have been well organized at the level of six transplantation centers since 1985. However, no country-wide structure existed to systematically monitor transplant outcomes and to coordinate multicenter studies during the post-transplant process. Each transplant program collected its own data, and no monitoring or auditing was performed. In 2006, several Swiss investigators from different disciplines decided to launch a prospective multicenter cohort project, the Swiss Transplant Cohort Study (STCS), aiming at a nationwide comprehensive and structured data collection in all solid organ transplant (SOT) recipients. After a 2-year set-up period the STCS started to be operative with the first patients enrolled in May 2008.

In a parallel development, a new transplantation law was enforced in 2007, requiring a mandatory life-long follow up of all transplanted patients in Switzerland. In a collaborative effort of the Swiss transplantation centers with the Federal Office of Public Health, the cohort ensures compliance with the requirements of the law.

Other national and international transplant registries have previously shown their value by generating a large body of knowledge in transplantation medicine. Examples are the Scientific Registry of Transplant Recipients (SRTR) in the US [1, 2], the Heidelberg-based Collaborative Transplant Study (CTS) in Europe [3, 4], the Australia and New Zealand Dialysis and Transplant (ANZDATA) registry [5], as well as the Spanish Resitra cohort [6, 7]. These large registries focus on specific biomedical factors but are often limited in regard to the integration of psychosocial and behavioral factors, infectious disease occurrence, immunologic factors and a variety of long-term outcome. Moreover, most of these registries are based on the willingness of the centers to contribute patient data. One of the aims of the STCS is to provide a complete nationwide long-term follow-up and embracing a comprehensive bio-psychosocial perspective in its data collection providing a unique instrument for comparative effectiveness research [8].

This article describes the rationale and design of the STCS, provides preliminary descriptive results and aims to integrate this new project into the environment of other existing observational studies.

The main objectives of the STCS are to:

- Record all SOTs within one unique database system in order to have a complete assessment of all patient-, transplant-, and center-specific activities in Switzerland.
- Prospectively collect high quality longitudinal clinical and laboratory data of transplant recipients at the Swiss national level, to evaluate the quality, effectiveness and efficiency of SOT in order to support patients, health care professionals and policy makers with informed decision-making.
- Implement a bio-bank sampling scheme to integrate biological and clinical information.
- Reflect the complexity of the post-transplant patient care in an appropriate data model and integrate this complexity into research hypothesis and methodology.
- Collect selected psychosocial and behavioral data at time of listing and during follow-up.
- Systematically capture relevant infectious diseases episodes.
- Record and periodically update specific risk profiles to reflect changes in disease- and treatment status.
- Assess determinants of poor short- or long-term outcome and allow studying alternative pathways that contribute to the understanding of patient – and allograft survival [6, 7, 9–11].

## Materials and methods

### Study design and setting

The STCS is a prospective multicenter cohort which was designed as a dynamic cohort study where SOT recipients move in and out as time progresses [12]. We define prospective in the sense that data definitions were made in agreement with the rationale of the study prior to the enrolment period, and that measurements are made in agreement with these definitions [13]. A version control strategy has been implemented that ensures data consistency over time, should changes or an updating of the data definitions become necessary.

A patient is considered as transplanted and therefore enrolled at the moment of transplantation, i.e. when the surgeon releases the clamps to start reperfusion of the allograft. For islets transplantation, we defined transplantation as the moment when the islets are injected into the recipient. At the time point of transplantation, the “patient clock” is set to zero initiating prospective follow-up of both the patient and the corresponding allograft(s). Any subsequent transplantation that may occur for a patient is prospectively registered within the patient-case system. Patient follow-up ends with death or definitive drop-out. Non-fatal graft failure does not truncate a patient’s follow-up (e.g. kidney transplant recipients).

### Participants

All recipients of SOTs in Switzerland are prospectively registered since May 2008. No particular eligibility or exclusion criteria exist for enrolment. Patients with grafts implanted before the start in May 2008 are not recruited retrospectively, unless such a patient presents for a re- or a second transplant. Tissue transplantations are not considered.

Switzerland has compulsory health insurance and transplantation is part of basic health care. Patients pay premiums with co-payments for medications.

### Patient-case system

**Fig. 1:**
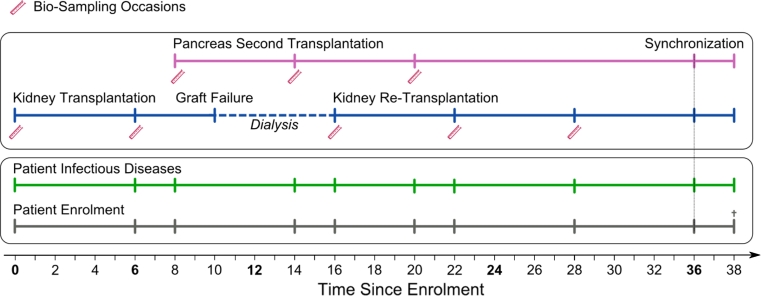
Organization of the Swiss Transplant Cohort Study patient-case system based on a hypothetical complex transplantation scenario

The core data structure of the STCS is the patient-case system, a framework that reflects the post-transplant patient process involving a multitude of information on patients and allografts, including function and interventions from transplantation until end of follow-up (Fig. 1).

The STCS patient-case system allows distinguishing data that accrue in relation to the patient from data related to the transplanted organ(s). We therefore define a “case” as any SOT of a given patient. A patient may have one or several cases, and one case can involve one, or more than one allograft. Each case nested within a patient has its own time axis and follow-up (“case clock”, Fig. 1).

Patient-data captures information which is of systemic nature and that relates to the patient, but not to the transplant itself. In contrast case-data captures information restricted to the allograft(s). The first case is the transplant event that leads to enrolment in our study. Later cases are termed re-transplants or second transplants. A re-transplant is a repetition of the same SOT after failure of the previous transplant; e.g. a kidney re-transplanted after loss of function of the previous kidney allograft. A second transplant refers to a subsequent transplantation of a different type of allograft; e.g. a pancreas transplantation following a successfully implanted kidney allograft. Each instance can either be a single or a double transplantation. Double transplantation refers to concomitant transplantation of two organs originating from the same donor.

Thus three classification layers can be distinguished: (1) the patient; (2) the SOT (classified into single or double/complex SOT and into first, second- or re-transplantation); (3) the implanted organ. E.g. both allografts of a kidney-pancreas double transplantation are treated as separate instances. Patients are usually classified by their first STCS (enrolment) transplantation.

**Fig. 2:**
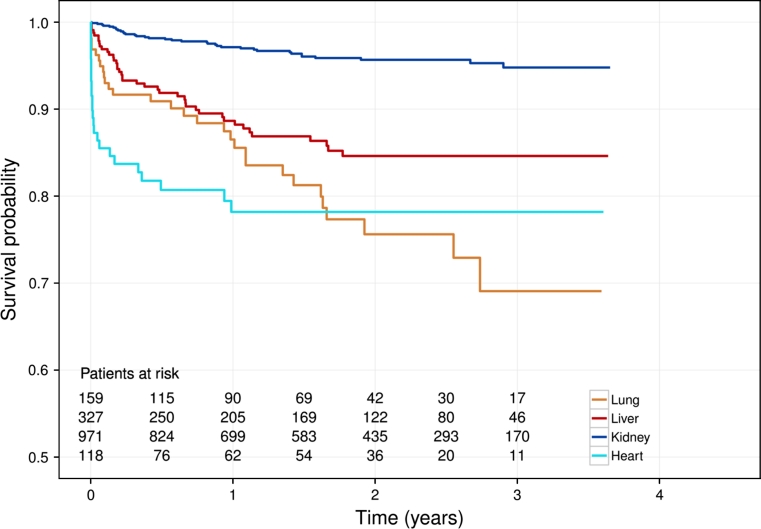
Overall patient survival by first transplantation in the Swiss Transplant Cohort Study (1.5.2008 until 30.09.2011)

Our patient-case system assigns unique patient and case identification numbers. Linkage of patient and case data allows reconstructing the transplantation process (Figs. 1 and 2) with longitudinal updating of both patient and case information, as well as capture of intermediate events. Donor-recipient linkage is ensured via the unique Swiss organ allocation number (SOAS-ID), which is generated within the national Swiss Organ Allocation System (SOAS) and is transferred to the STCS. Donor data specification is detailed in the Appendix (in ESM).

## STCS cohort data

### Data collection schedule

The baseline and follow-up data collection schedule is part of the STCS patient-case system (Fig. 1). After transplantation, all patients are mandatorily followed in their respective transplant centers. After baseline assessment, STCS follow-up assessments take place at 6-, 12 months and yearly thereafter. In the case of a second or re-transplantation, we perform three extra assessments of the new case at baseline, 6-, and 12 month and we update the patient data in regard to the new case. After completion of these three extra visits, the schedule is synchronized to the initial patient visit schedule (“patient clock”).

Biological samples are collected in relation to the case at baseline, and at 6-, and the 12-month visits. Psychosocial assessments are performed at time of listing, at 6-, 12 months and yearly thereafter along the “patient clock”. Specific data forms exist to track samples and to record infectious events, death and drop-out.

### Data definitions and measurements

On the patient-level, data collection extendedly focuses on psychosocial questionnaire (PSQ)-, infectious disease-, cancer, and causes-of-death data; on the case-level on bio-sampling, organ function, immunologic events and causes of graft failure. A separate repository exists for medication data, including induction, maintenance immunosuppression; infectious disease prophylaxis and a selection of other relevant drugs. Where appropriate, data are collected longitudinally and are thus updated over time. All details on STCS data including data definitions and measurements are given in the Appendix (in ESM).

## Data processing, management and data quality assurance

The local transplant coordinators have full access to the SOAS and they are informed about all listings and transplantations. They work in close collaboration with the STCS local site data managers (LDM) and provide information about enlisted patients and all recent transplantations performed at their center.

Local site data managers are responsible for data collection pertaining to a certain follow-up period (e.g. baseline, 6 month, yearly). All LDM have an STCS local transplant physician at their side for support in data access and medical content support. All data are entered into patient- and organ-specific online case report forms (CRF) and all data are electronically transferred to a central database system.

Standard operating procedures (SOPs) establish the working standards, address legal aspects of consent handling, bio-sampling and on updating of the STCS infrastructure.

Under the mandate of the Swiss federal office of public health [14], nationwide data quality audits are performed to improve data quality and enhance between-center standardization.

The STCS implemented an endpoint committee that reviews all death registered within the STCS database on a regular basis. Causes of death are determined at the site by two physicians independently. Disagreement in coding is resolved by consensus. We code causes of death according to the US Centers of Disease Control and Prevention (CDC) system based on immediate and underlying causes of death [15].

The database is maintained by the Division of Medical Information Sciences, University Hospitals of Geneva. The system is entirely based on “attribute-values” entities [16] that avoids to use conventional relational techniques. Attribute-values approaches are perfectly adapted to answer evolving data models and heterogeneous data representation [16].

## Structure and organization

The STCS is a scientific project with a primary interest in clinical research and with a strong secondary interest in the control of quality of care requested by the Swiss law on transplantation. The study is investigator-initiated. All participating centers and epidemiologists contributed to the design of the STCS. The STCS does not pursue financial interests. The institutional review boards of all transplanting centers approved the participation in the STCS.

The STCS is operationally led by an Executive Office (EO). The STCS steering committee, called the board of representatives (BOR) includes representatives from all centers. Various working groups provide expert advice. The STCS scientific committee, assembled from representatives of all participating centers and the various medical specialties involved in transplantation, covers all aspects regarding the conduct of scientific research projects nested within the STCS. The STCS regularly reports to the Swiss federal office of public health [17, 18] in order to comply with the national requirements on quality control.

The conduct of the STCS was approved by the independent ethic committee of each Swiss transplant center. Written information about the STCS is distributed to patients during listing. To obtain the full cohort data including bio-samples, patients are asked to provide written informed consent while listed or latest at the time of enrollment. For patients who deny consent, the law mandates collection of a set of mandatory data (“minimal dataset”) involving a restricted number of transplant-relevant baseline and endpoint data [19].

## Results

### First descriptive data for the period May 2008 until end of 2011

Between May 2008 and end of 2011, all six STCS centers recruited 1,677 patients that underwent 1,721 transplantations involving a total of 1,800 implanted organs (Tables 1 and 2). 93% of all SOT recipients consented to STCS participation. The monthly patient recruitment rate varied between 10 and 60 patients over time. By end of December 2011, we had recruited patients with e.g. 981 single kidney, 346 liver, 164 lung, 119 heart, and 27 islets transplantations. The classification of these patients is according to the first STCS (enrolment) transplantation. The most frequent enrolment double transplantations were kidney-pancreas (n=41) and kidney-liver (n=16). 10% percent of all patients underwent re-transplantation and 3% had a second transplantation, either in the past prior to the initiation of STCS or during STCS follow-up. We prospectively registered 15 different transplantation scenarios, including seven single, and eight double or triple transplantation scenarios.

**Table 1:** Selected patient baseline information according to the transplantation that led to Swiss Transplant Cohort Study enrolment (1.5.2008 until 31.12.2011)

	Heart (n= 118)	Single kidney (n= 971)	Liver (n= 327)	Lung (n= 159)
Age, median (IQR)	52 (40–60)	53 (41–62)	54 (43–60)	54 (34–60)
Male gender [n, (%)]	87 (74)	629 (65)	207 (63)	77 (48)
Pediatric [n, (%)]	10 (9)	39 (4)	28 (9)	7 (4)
Smoking [n (%)]				
Current	1 (1)	124 (14)	71 (25)	1 (1)
Past smoking	65 (62)	245 (28)	86 (30)	66 (46)
Never smoked	30 (29)	423 (48)	85 (30)	63 (44)
Answer refused	0	6 (1)	3 (1)	0
Missing data	8 (8)	79 (9)	37 (13)	9 (6)
Pre-transplant working status* [n (%)]				
>80%	21 (20)	134 (15)	61 (22)	11 (8)
51–80%	6 (6)	78 (9)	19 (7)	9 (6)
21–50%	11 (11)	146 (17)	22 (8)	24 (17)
1–20%	4 (4)	41 (5)	9 (3)	8 (6)
0%	54 (52)	383 (43)	125 (44)	82 (57)
Answer refused	1 (1)	15 (2)	7 (2)	0
Missing data	8 (8)	87 (10)	38 (13)	9 (6)

* Pre-transplant working status categories in percent represent full- or part-time working capacity. >80% is considered as full-time working capacity

**Table 2:** Overview of implanted organs in 1,677 patients enrolled in the Swiss Transplant Cohort Study (1.5.2008 until 31.12.2011)

	Overall	Organ from first Tpx	(%)	Organ from re-Tpx	(%)	Organ from second-Tpx	(%)
Kidney	1,067	885	82.9	165	15.5	17	1.6
Liver	363	321	88.4	33	9.1	9	2.5
Lung	166	154	92.8	9	5.4	3	1.8
Heart	119	117	98.3	2	1.7	0	0.0
Islets	33	11	33.3	12	36.4	10	30.3
Pancreas	50	43	86.0	1	2.0	6	12.0
Small bowel	2	1	50.0	1	50.0	0	0.0
Total	1,800	1,532	85.1	223	12.4	45	2.5

Note: Allografts from simultaneous double or multiple transplantations (e.g. kidney-pancreas double transplantation) were re-distributed into the corresponding organ categories

A total of 34 % of all single kidney transplants originated from living donation, of which 55% were from living-related, and 45% from living-unrelated donation. Thirteen liver transplantations arose from living-related and 7 from living-unrelated donation.

The median follow-up duration was 1.7 years (IQR 0.7–2.6) for the stated period. Overall, 136 patients died, 102 had allograft failures and four patients were lost to follow-up; three moved away and one did not respond to repeated contact. Figure 2 shows the overall patient survival stratified by the four most frequent STCS first transplantations.

**Fig. 3:**
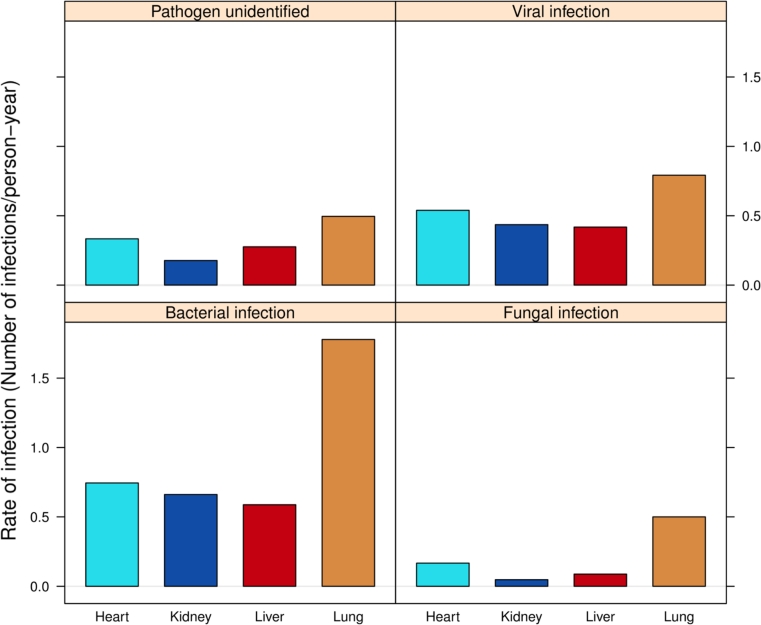
Rate of proven infectious diseases by type of transplantation and pathogen in the Swiss Transplant Cohort Study (1.5.2008 until 31.12.2011)

We observed 4,385 infection episodes in our patient population during the observation period. 1,048 patients (62%) showed at least one bacterial, viral, fungal or parasite infection episode. 521 (31%) had at least one proven bacterial or fungal disease, or a viral syndrome (Table 3, Appendix in ESM). Figure 3 shows the rate of proven diseases due to the mentioned pathogen groups in patients stratified by their STCS first transplantation.

**Table 3:** Summary of collected data on the occurrence of infection episodes in the Swiss Transplant Cohort Study (1.5.2008–31.12.2011)

Patients	1,677
Patients with any infectious event [n (%)]	1,048 (62%)
Patients with any proven infection or viral syndrome	521 (31%)
Average number of infectious events in subjects with at least one infection, median	4.17
Average number of proven infections or viral syndrome in subjects with at least one such episode	1.76

In consenting patients, we moreover harvested 3,630 plasma-, 3,570 viable cell—and 1,663 extracted DNA samples. Samples could be obtained in 98% of all consenting patients (see sampling scheme Fig. 1).

Table 4 shows the number of case-report forms where the mandatory data were complete, partially complete or missing. Overall, 96.6% of CRFs with a closed follow-up period were complete. The highest number of missingness was observed in liver transplantation.

**Table 4:** Completeness of patient and organ baseline and follow-up information in the Swiss Transplant Cohort Study (1.5.2008 until 31.12.2011)

CRF	CRF completeness status			Total
	CRF completed*	CRF partially completed**	CRF empty	
	n (%)	n (%)	n (%)	
Patient baseline	1,656 (98.7)	1 (0.1)	20 (1.2)	1,677
Patient FUP	3,240 (96.1)	0 (0)	133 (3.9)	3,373
Patient stop	134 (95.7)	6 (4.3)	0 (0)	140
Heart baseline	116 (97.5)	3 (2.5)	0 (0)	119
Heart FUP	175 (97.8)	0 (0)	4 (2.2)	179
Islets baseline	32 (97.0)	1 (3.0)	0 (0)	33
Islets FUP	55 (87.3)	3 (4.8)	5 (7.9)	63
Kidney baseline	1,056 (99.0)	11 (1.0)	0 (0)	1,067
Kidney FUP	2,227 (97.4)	11 (0.5)	49 (2.1)	2,287
Liver baseline	330 (90.9)	33 (9.1)	0 (0)	363
Liver FUP	575 (91.3)	10 (1.6)	45 (7.1)	630
Lung baseline	166 (100.0)	0 (0)	0 (0)	166
Lung FUP	254 (94.1)	2 (0.7)	14 (5.2)	270
Pancreas baseline	50 (100.0)	0 (0)	0 (0)	50
Pancreas FUP	154 (93.3)	0 (0)	11 (6.7)	165
Small bowel baseline	2 (100.0)	0 (0)	0 (0)	2
Small bowel FUP	3 (100.0)	0 (0)	0 (0)	3
Over All	10,225 (96.6)	81 (0.8)	281 (2.7)	10,587

According to standard operating procedures, a 90 days’ time span is allowed for data capture and entry. CRFs of patients who died or who lost their graft within a follow-up period were removed for this analysis. 176 CRFs were removed from the analysis since the follow-up period was not yet completed

* Completed: >80% of all mandatory data captured

** Partially completed: at least one content entry captured

*CRF* case-report form, *FUP* follow-up

## Discussion

The STCS is a novel prospective cohort study that comprehensively monitors all SOT activities in Switzerland. The current experience shows that the STCS has become an operating cohort that allows highly complete and adequate capture of important transplant-related events. Transplant scenarios of any complexity can be reflected in detail along with the collection of psychosocial, infectious disease and transplant-relevant outcome data.

### What makes the STCS unique among the existing registries?

In the past, large collaborative studies have provided important knowledge in the field of transplantation. These include the Heidelberg-based Collaborative Transplant Study (CTS). This registry is based on the voluntary cooperation of more than 400 transplant centers in 45 countries, which has included more than 4,00,000 recipients of kidney, heart, lung, liver, and pancreas transplantations [3]. In the US, national data on solid organ transplantations are collected through the Organ Procurement and Transplantation Network (OPTN) [20]. The National Institute of Health furthermore sponsors the “Adult to adult living donor liver transplantation cohort study” (A2ALL), including data from 9 US transplant centers. The International Registry for Heart and Lung Transplantation (ISHLT) provides information on the thoracic organ transplant experience around the world [21]. Since 1968 the European Liver Transplant Registry (ELTR) collected data regarding over 71,000 liver transplantations performed in 137 European centers [22]. The Australia and New Zealand Dialysis and Transplant (ANZDATA) [5] and the Australian and New Zealand Cardiothoracic Organ Transplant Registry (ANZCOTR) [23] are comprehensive, population based registries.

All these large cohorts suffer to some extent from the heterogeneity of clinical follow-up data and none implemented clear-cut definitions of transplant related outcomes such as rejection and infections. Most registries provide their data on a voluntary basis and data monitoring activities according to a priori defined quality standards are limited. Moreover there are differences in immunosuppressive regimens, in prophylaxis and therapeutic infectious strategies, differences in availability of patient care due to large distances between transplant centers and patient homes, as well as differences between centers within and between different countries.

Many registries and large cohort studies focus only on one type of solid organ transplant population (e.g. USRDS, ELTR, A2ALL, ANZDATA) or are limited to thoracic organs (ISHLT), thus limiting the options for comparisons among different transplant populations. Some registries include several types of organ transplants (e.g. CTS), but are limited to centers who volunteer in participation [3, 24] with the uncertainty about potential selection and information bias. Further issues may relate to data quality: a comparison of OPTN/SRTR with A2ALL supported this hypothesis and claimed center-specific data monitoring to substantially improve the data [25].

Findings from large registries often result in interesting hypotheses, which in turn need to be validated in subsequent studies because of the lack of systematic collection of recipient serum and cell samples, and/or prospective outcome data [26]. None of the cohorts monitors prospectively from time of listing the life-long post-transplant course by means of clinical and selected patient reported outcome data.

The STCS is a unique and novel prospective, comprehensive cohort study that attempts to fill these gaps. Indeed the STCS follows by law all transplanted patients at the Swiss national level, therefore preventing patient selection processes and potential selection bias. Furthermore the STCS was designed to collect ample clinical, psychosocial, immunologic, infectious diseases, metabolic and cancer data, paralleled by the harvesting of genomic DNA, plasma and live peripheral blood mononuclear cells in consenting patients (currently 93%). In addition database cross-matching with the Swiss Organ Allocation System (SOAS) ensures complete patient enrolment and transparently shows all solid organ transplantation activities in Switzerland. Also the linkage with the Swiss Monitoring of Potential Donors (SwissPOD) study potentially allows access to an extended range of donor data. The relatively small geographic characteristics of Switzerland as a country with short distances allow adequate long-term follow-up for almost all SOT recipients across the country. Working groups in all transplant-related medical specialties continuously cooperate on data definitions and on homogeneous data collection. Moreover these working groups defined appropriate diagnostics tools (e.g. CMV viremia detection), as well as prophylactic and therapeutic strategies for infectious events. A well-recognized example for the success of Swiss cohorts based on similar characteristics is the Swiss HIV cohort study (SHCS) [8, 27] which has become a worldwide reference in HIV research.

A key strength of the STCS is its rigorous longitudinal data structure. Changes in exposure to risk factors (e.g. smoking, medication non-adherence) or medication use are registered, and in parallel changes in the patient’s health status, physical functioning, work ability or organ function are longitudinally updated. It is one of the priorities of the STCS to focus on chronic disease burden beyond the mere transplant outcomes, and to reflect the complexity of the post-transplant process in the population of Swiss SOT recipients. Along an inherently consistent data structure, the STCS priorities are high-data quality and minimization of attrition by standardized data management processes (SOP’s) and site monitoring for data quality. In line with this cohort architecture, the STCS endorses the reporting according to the STROBE statement [13].

The STCS allows for a comprehensive capture of all transplanted patients on the country level. This is only possible since the consent barrier for minimal data collection can be overruled by law in Switzerland, with the potential to address scientific hypotheses based on real-life and long-term data. Future STCS projects will provide novel insight on e.g. infectious disease occurrence, comparison of STCS patient—and organ survival with existing registries, the influence of center effects and genetic association studies.

### Limitations of the STCS and sources of bias

Compared to other national and international large registries, the STCS is a relatively small-sized cohort study, with the limitation of small numbers particularly in uncommon transplant scenarios. However the restricted size was an advantage for the creation of the described cohort. In larger countries it would have been very difficult to create a nationwide cohort initiative with a comprehensive and high quality data structure, bio sampling and with the possibility of consistent longitudinal data structure.

All routine laboratory and sampling procedures are processed in local transplant centers, what may be seen as a limitation. However, standards, laboratory methods, units and detection limits differ only slightly between these local laboratories and will therefore likely be small and not lead to important center effects regarding patient care.

## Conclusion

The Swiss Transplant Cohort Study (STCS) is a new prospective collaborative multicenter cohort, which systematically monitors all SOTs on the Swiss national level, and puts into place a novel patient-case system that allows re-constructing and operationalizing all post-transplant scenarios, thus reflecting the complexity of the post-transplant process. The unique geographic characteristics of Switzerland are an advantage in regard to high quality long-term prospective observation. The comprehensive clinical data aligned with bio sampling makes the STCS unique as a longitudinal transplant cohort. It is suggested that the longitudinal nature of the study design provides the basis for advanced modeling of the interplay of biological, psycho-social and system factors with the potential to improve transplant outcomes.

## Electronic supplementary material

Below is the link to the electronic supplementary material.

Supplementary material 1 (DOC 371 kb). Supporting Information: Additional Supporting Information may be found in the online version of this article. Supporting material is available as supplementary appendix on data definitions (“appendix”)

## Abbreviations

STCS: Swiss Transplant Cohort Study
SOT: Solid organ transplant
ID: Infectious disease
PSQ: Psychosocial questionnaire
