# Supplementary material for: Design and methodology of the Swiss Transplant Cohort Study (STCS): a comprehensive prospective nationwide long-term follow-up cohort
Source: Eur J Epidemiol. 2013 Apr 2;28(4):347–55. doi: 10.1007/s10654-012-9754-y (PMC3653038; doi:10.1007/s10654-012-9754-y)
Supplement: Supplementary file 1 — Supplementary material 1 (DOC 371 kb). Supporting Information: Additional Supporting Information may be found in the online version of this article. Supporting material is available as supplementary appendix on data definitions (“appendix”) [file 10654_2012_9754_MOESM1_ESM.doc]

**Appendix**

Mandatory (minimal) data to be collected by law are characterized with an asterisk (*).

**Patient pre-transplant diseases, co-morbidity and incident diseases following STCS enrolment**

*Prevalent and incident diseases*

This section generally provides information on pre-transplant disease history, prevalent (co-morbid) and incident diseases. Prevalent indicates co-morbidity conditions present at baseline i.e. present prior to the index transplantation leading to STCS enrolment. Incident relates to diseases that occur during the prospective post-transplant follow-up in individuals known to be “disease-free” at baseline. Given the context, incident either refers to future disease events (e.g. acute rejection, occurrence of post-transplant lymphoproliferative disease [PTLD]) or to disease events that are defined to become relevant by the initiation of treatment (e.g. initiation of pharmacologic treatment for diabetes type 2 or dyslipidemia after failures of conservative treatment).

We collect all past transplantations* with the corresponding transplantation dates prior to STCS inclusion, together with the information on past immunosuppressive medication use. The actual enrolment transplantation and all following transplantations are registered within the STCS patient-case system. Cardiovascular events (using standard criteria for coronary heart disease [e.g. myocardial infarction [1]], ischemic [2] or haemorrhagic [3] cerebral vascular stroke, peripheral vascular events [4]), left ventricular dysfunction (defined as an ejection fraction (EF) < 30%), or venous thrombosis/pulmonary embolism occurring prior to STCS enrolment or during prospective follow-up are registered.

Prevalent or incident metabolic, endocrine or kidney diseases are collected as treated hypertension [5], diabetes mellitus (DM) type 1 and type 2 according to WHO definitions [6], dyslipidemia [7], and chronic kidney disease with or without need for renal replacement therapy. DM type 2 and dyslipidemia are collected only if they require pharmacologic treatment. Data collection for malignancies * includes any occurrence of skin cancer, specified as squamous cell carcinoma, basal cell carcinoma or melanoma. For cancer other than skin, we primarily defined common and transplant-relevant cancer types and sites such brain, breast, cervix/endometrium/adnex, colon, Kaposi’s sarcoma, kidney, leukaemia, post-transplant lymphoproliferative disease (PTLD), liver, lung, lymphoma, multiple myeloma, bladder and prostate cancer. For each malignancy, we register and update the disease-status at baseline and during the prospective follow-up period as new detection, relapse following treatment, persistence or progression, or in remission. We moreover record a limited number of past infectious events during the baseline assessment: past *M. tuberculosis* infection, Aspergillosis, S.aureus (MRSA), ESBL-producing Gram-negative bacilli colonization, or parasitic infection. Post-transplant infectious diseases are detailed further below. Other relevant events include major non-transplant related surgery, osteoporosis with bone fracture, prior or future occurrence of neutropenia (count <500/mm3 [8]), and pregnancy outcome in female recipients (live birth, abortion or stillbirth).

*Clinical Assessments*

From physical examination we record data on measured body weight, height, systolic and diastolic blood pressure measured according to standard criteria in a sitting position whenever possible [9], and ethnicity (Caucasian, African or African-American, Asian, or “other ethnicity”). All assessments but ethnicity are repeated at each regular cohort follow-up visit.

*Routine laboratory assessments*

The following laboratory parameters are measured at baseline and during each STCS scheduled follow-up visit: serum creatinine*, total cholesterol (TC), HDL-cholesterol (HDL-C), LDL-cholesterol (LDL-C), and plasma glucose (random or fasting). In diabetic patients, the HbA1c value is collected. Each reading is recorded together with the corresponding sample collection date. Lab assessments are scheduled according to the STCS visit schedule. If lab assessments are missing, the closest assessment to the cohort visit from hospital records is entered. The AB0 blood group* is recorded at baseline. HLA tissue typing, immunologic assessments and organ-specific measurements are stored as organ-specific data, as further detailed below.

*Medication data*

Medication data are recorded as patient-related data. The only exception relates to treatment of allograft rejections that are recorded along with each specific rejection episode. We defined drug exposure periods with start - and stop-dates. Medication data are updated during each cohort visit occasion and at the time point of re-or second transplantation. We collect drug substances, drug classes or treatment procedures for induction treatment* (basiliximab, daclizumab, thymoglobulin, ATG, ATGM, OKT3, campath, rituximab, IVIG, or the use of plasmapheresis), for maintenance immunosuppression* (cyclosporin, tacrolimus, everolimus, sirolimus, glucocorticoid, MMF, EC-MPA, azathioprine), for infectious disease prophylaxis (valganciclovir, valaciclovir, trimethoprim-sulfamethoxazole, atovaquone, pentamidine, voriconazole, fluconazole, beta-lactame antibiotics, cephalosporin, quinolone, metronidazole, posaconzaole, caspofungine, ganciclovir, aciclovir) or a number of classes of “other drugs” that were judged to be of interest to study the post-transplantation process (insulin, oral anti-diabetic drugs, statins, ACE inhibitor, angiotensin receptor blocker, calcium channel blocker, beta-blocker, anticoagulation therapy, or platelet aggregation inhibitor use). For tacrolimus and MMF we moreover collect whether the original or a generic drug preparation was used.

*End of follow-up data*

The STCS follow-up period ends with death or definitive drop out. All stop data* are recorded with the corresponding dates*. Causes of death* are determined according to the WHO definition of immediate causes of death and underlying causes of death. Causes are coded by ICD-10 [10]. The immediate cause of death indicates the process or complication that directly leads to death. The immediate cause is the ultimate consequence of the underlying cause of death. The underlying cause indicates the disease that initiated the train of morbid events leading directly to death [11]. A patient who dies of pulmonary embolism caused by metastatic cancer illustrates the immediate and underlying cause of death, respectively. Appendix table 1 shows the list of predefined immediate and underlying causes of death using an extension of the causes of death determined by Budion et al [12]. In the case of a patient’s drop out, we collect the reasons for drop out* (moved away, non-response to several invitations, too sick or handicapped to continue, patient wish to discontinue).

**Infectious disease (ID) data**

Infectious disease (ID) data have been standardized and are collected and validated by a transplant ID specialist at each transplant center. We collect ID data as events that occur as distinct, but within-patient repeatable points in time. The data collected includes the date, type of pathogen (species), the type of infection (i.e. bacterial colonization or infection; possible, probable or proven fungal infection; asymptomatic viral replication or symptomatic viral disease etc), the site of infection, as well as corresponding treatment and the potential that a given ID event might be a donor-related infection. Proven bacterial infection is defined by the combination of: isolation of a bacterial pathogen plus compatible clinical signs and/or symptoms plus specific antibiotic treatment. Proven viral infection is defined by detection of virus replication with the corresponding pathology in biopsy tissues. Viral syndrome is defined by the detection of virus replication and non-organ specific clinical symptoms (fever). Proven fungal infection is defined by (I) histopathology of a specimen obtained by biopsy with hyphae, yeast cells or melanized yeast-like forms accompanied by tissue damage, or (II) culture of a mold, “black yeast” or a yeast from a normally sterile plus clinically and/or radiological signs consistent with an infectious disease process, or (III) PCR-based detection of a yeast/mold in a sterile tissue, or (IV) positive fungal blood culture, or (V) Cryptococcal antigen in CSF, or (VI) detection of Pneumocystis jirovecii by cytology/microscopy/histology/elevated PCR accompanied by clinical symptoms.

The definitions made by the STCS ID group are based on internationally accepted definitions such as the European Conference on Infections in Leukemia (ECIL) [13] and Infectious Disease Society of America (IDSA) [14] definitions for bacterial, fungal and viral infections. Recurrent events such as asymptomatic viral replications, as well as bacterial colonization of a given site are documented once per follow-up period. Standardized and homogenous registration of ID events according to these definitions is assured by regular training, using typical cases of ID events that are shared inside the STCS ID working group. Regular contact between the six transplant ID physicians allows feedback and discussions for particularly difficult cases.

**The STCS Psychosocial Questionnaire (PSQ)**

Selected psychosocial and behavioral variables are collected as patient reported outcomes at regular time points during life-long follow up using the STCS psychosocial questionnaire (PSQ). This is a self-report instrument that integrates validated instruments as well as items from other cohort studies or nationwide surveys.

Two consistent sets of the PSQs were developed: A pre-transplant and a post-transplant follow-up questionnaire. Each questionnaire is available in four languages (English, French, German and Italian). Backward-forward translations have been done using standard cultural-sensitive translation protocols [15].

Patients on the waiting list are contacted by regular mail to fill in the PSQ (during the informed consent process). We use addressed and stamped envelopes to simplify the response process. Alternatively, the patients return the completed PSQ to the sites during pre-transplant visits. Post-transplant follow-up PSQ assessments are done by regular mail or during site follow-up visits. A written reminder system has been implemented if the participant does not respond within 6 weeks for waiting list patients and 10-14 days for post-transplant patients.

*Socio-demographic variables*

The level of education is assessed by an item derived from the Swiss Health Survey (SHS) and is defined as the highest completed level of education (no school, mandatory school, apprenticeship, bachelor, higher professional education, higher technical or commercial school, university, other) [16]. Marital status is also derived from the SHS [17] and assessed as single, married/living together, widow/widower, divorced/separated. Employment status refers to the two items professional status and working ability. Professional status is assessed in correspondence to the SHS [17] as the current regular occupation or last held professional position (self-employed, working in a relative’s firm or business, apprentice/trainee, director/manager, middle/lower staff, employee, houseman/-wife, disability rental, pension, student, or other). Working ability, an item derived from the Swiss HIV Cohort Study (SHCS) [18], is defined as the percentage of time spent in professional earning activity during the past 6 months (pre-Transplant PSQ) or since transplantation (post-Transplant PSQ) (> 80%, 51%-80%; 21%-50%, 1%-20%, 0%). For subjects responding 0%, the reason is requested (i.e. housewife-/ man in your own home, in education, retirement pensioner, illness, unemployed, invalidity pensioner). Finally, the socioeconomic status using income as proxy as derived from the SHS [16]. We ask how much money the patient and other household members have available in total per month (i.e. < 4500 CHF, 4501-6000 CHF, 6001-9000 CHF, >9001 CHF).

*Behavioral variables*

Selected behavioural variables involve medication adherence, smoking, harmful substance use and sun protection.

Medication adherence is assessed by two items derived from the Swiss HIV cohort study (SHCS) [19;20] which were taken from the BAASIS [21], an instrument specifically developed for transplant adherence research. More specifically, the two adherence items used refer to two of the four dimensions (taking adherence and drug holidays) of medication taking behavior. The timing dimension and reduction of dosage which are part of the BAASIS are not assessed. Item one involves adherence by asking patients ‘How often did you miss a dose of your medication (pre-Transplant) or your immunosuppressive drugs (post-transplant) in the past 4 weeks?’ (never, once a month, once every two weeks, once a week, more than once a week, every day). The second item addresses drug holidays (“did you miss more than one dose of medication in a row?”). Adherence to medication or immunosuppressive drugs is defined according to the number of missed doses as used in previous studies [19]. Predictive validity of this medication adherence measure has been shown in the HIV population with regard to viral rebound [19]. Furthermore, the instrument showed fair diagnostic values with sensitivity of 87.5% and specificity of 78.6% when compared to prospective one year virologic failure in a sample of 133 patients with HIV [22].

The smoking behaviour is assessed by an item used in the SHCS [23] or other large cardiovascular cohort studies (ref). During the pre-transplant assessment we ask “Do you smoke?” with answer options; current, past (stopped < 1y ago), past (stopped > 1y ago), and never. The post-transplant smoking behaviour involves the question ‘Have you smoked during the past 6 months?

Capture of sun exposure data and sun protection behaviour implemented in the PSQ follows an abridged version of the suggestion of Glanz et al. [24]. The items involve first occupational sun exposure and sun exposure during leisure time (in categories of hours per day), and second sun protection behaviour by the patient self-reported use of sunscreen and wearing of hats.

*Psychosocial wellbeing*

With each PSQ assessment, we record the depressive symptoms subscale of the Hospital Anxiety and Depression Scale (HADS-D) [25-27]. The HADS-D is a non-disease specific self-report non diagnostic screening instrument developed for assessing the cognitive symptoms of anxiety and depression in medically ill populations. It has been widely used and has been well validated as a screening instrument for anxiety and depression in the general medical population [26;28;29] and to a lesser degree in the context of liver [30;31], lung [30;32] and kidney transplantation [33]. The scale consists of two subscales with a total of 14 items: seven items measuring anxiety and seven items assessing depressive symptomatology. Items are rated from 0 (not at all/hardly at all) to 3 (most of the time/very definitely).

We use two items to rate patients sleep quality. The first item addresses sleep quality, the second daytime sleepiness. These are two opposing complementary aspects of the sleep phenomenon [34-36]. Sleep quality is assessed by 1 of 4 items measuring sleep from the Kidney Disease and Quality of Life questionnaire (KDQOL-SF™ 1.3) developed for individuals with end stage renal disease [37;38]. This item was used in the DOPPS [39] and showed predictive validity for mortality in hemodialysis patients (cutoff ≥6). More specifically, patients are asked ‘on a scale from 0 to 10, how would you rate your sleep overall?’ and their answer is scored on a 10 point scale from 0 (very bad) to 10 (very good). Daytime sleepiness will be assessed by asking patients ‘on a scale from 0 to 10, how would you rate your daytime sleepiness overall?‘ Patients will score this item on a 10 point scale from 0 (not at all sleepy) to 10 (very sleepy) [40].

*Perceived health status:*

Literature searches indicated that SF-6D and the EuroQol (EQ-5D) were suitable candidate instruments for implementation in the STCS. Both showed widespread use and validity in the transplant literature [41-45]. It seems that the SF-6D does to a lesser extent describe health states at the lower end of the utility scale but is more sensitive than EQ-5D in detecting small changes towards the top of the scale [46;47]. That means that the SF-6D is rather sensitive to detect severe health states changes. Since we plan long-term follow-up of STCS patients, we expect differences to happen rather at the lower end of the utility scale and therefore we decided to implement the EuroQol (EQ-5D).

**General organ-related data**

General organ related data cover immunology and HLA tissue typing, donor and recipient infection serology data, common donor data, data on the transplantation procedure and peri-operative care, information on each biopsy performed at baseline and/or during patient follow-up.

We record a broad line-up of pre-transplant immunologic assessments that are performed at each study centre. HLA tissue typing involves the donor and recipients HLA A, B, and DR phenotyping*. Each donor-recipient T and B cell CDC cross-match study is recorded according to the interpretation of the local centre lab (positive or negative). For panel reactive antibody (PRA) studies against MHC class I and class II proteins, we record the peak values and the latest value in per cent prior to transplantation. Moreover, anti-HLA class I and class II antibody screening tests and the results as well as tests to detect anti-HLA class I and class II donor-specific antibodies (DSA) are collected. For each test, we collect the analytic tool used i.e. CDC, ELISA, or flow cytometry (FCM) based on Luminex or other methods. Post-transplant immunologic monitoring is performed at the discretion of transplant centre and covers the same tests listed above for the pre-transplant immunologic assessments.

We determine the pre-transplant (baseline) infection serology status of the donor and the recipient. For each test, we store the interpretation (positive or negative) according to the manufacturer’s criteria and the date of assessment. Hepatitis B involves the presence of Hbs-Ag, anti-Hbs antibody, anti-Hbc antibody. In cases of an active infection, the recipients HBV DNA viral load is recorded in international units/ml and copies/ml. For hepatitis C, we collect the result of the anti-HCV antibody test, and the recipients HCV RNA viral load in international units/ml and copies/ml in the case of active HCV infection. For CMV, EBV, toxoplasmosis, herpes simplex, HIV1 and HIV2, and VZV and syphilis (treponemal antibody test), we collect the serological screening results analogously.

All lab studies including immunologic testing, determination of serologic markers and routine lab measurements are performed by the local site laboratories.

For each allograft biopsy performed at baseline or during follow-up, we collect the biopsy date, the indication (diagnostic biopsy for suspected rejection or graft disease, protocol or surveillance biopsy including time zero biopsy), and the biopsy identification number.

*Donor data*

In addition to serological screening and immunological testing, we record the donor birthdate and gender, the donor type* (brain dead donor, donation after cardiac death, living related donor, living unrelated donor), the donor cause of death* (cerebral trauma, cerebral haemorrhage, cerebral disease, cerebral tumour, suicide, anoxia, other), and donor AB0-blood group. Living related donation* refers to donation among genetically related offspring i.e. siblings or parents. The STCS system was designed to enable linkage of the donor data from the Swiss Organ Allocation System (Swisstransplant, SOAS [48]) with each organ transplanted within the STCS.

*Data related to the actual transplantation*

Data involve the listing date* and the transplantation date*, the hospital admission and discharge data during which the current transplantation took place, and the cold ischemia times.

**Specific organ-related data**

*Kidney*

The comprehensive list of the predefined end-stage renal diseases leading to renal transplantation is provided in appendix table 2. For each patient we collect, whenever possible, the native renal disease* and the date of disease diagnosis in the past, whether the end-stage renal disease was histologically proven, and the type and duration of renal replacement therapy. Furthermore, we collect data on previous native or allograft nephrectomy and pre-transplant sensitizing events such as pregnancies, blood transfusions prior to transplantations. The renal transplantation case moreover involves the registration of the transplantation side and whether a dual (kidney-kidney) or single renal transplantation was performed.

After kidney transplantation we observe the date and cause of graft failure* if present (appendix table 3). The date of allograft failure alive is defined as the initiation time point of renal replacement therapy for allograft failure. We collect all allograft biopsies that accrue during follow-up and classify each according to the BANFF system [49;50]. We moreover code the clinical interpretation* (AHR (C4d pos [51]), ACR interstitial C4d positive, ACR interstitial C4d negative, ACR vascular C4d positive, ACR vascular C4d negative, Mixed ACR and AHR, borderline tubulitis, glomerulitis and/or peritubular capillaritis) and whether a rejection was clinical or subclinical. Treatment of rejection episodes* is indicated with the drug combination used. In addition to acute and chronic immunologic events, we collect renal allograft diseases of potential non-immunological origin (table 3). Transplant-relevant and -related urologic and surgical complications are registered (for example urinary leak or outflow obstruction, renal artery stenosis/kinking etc, table 4).

We monitor renal transplant function using several repeatedly measured variables. Early oligouria or anuria* was defined as less than 500 ml urinary output within the first 24 hours of transplantation and delayed graft function (DGF)* was defined as need for dialysis beyond day 7 of transplantation. We finally collect at each follow-up visit the urinary protein to creatinin ratio* in mg/mmol and BKV viremia*, if present, in copies/ml.

*Heart*

The comprehensive list of native diseases leading to heart transplantation has been pre-specified (appendix, table 3). Related to the native heart disease, we collect, whenever possible, the date of disease diagnosis in the past and the history of cardiac interventions (valvular surgery, coronary artery bypass grafting (CABG), pacemaker, implantable cardioverter defibrillator (ICD), cardiac re-synchronization therapy (CRT) device without ICD or CRT with ICD implantation, Ventricular Assist Device (VAD)). We collect baseline pre-transplant data on the duration of VAD support prior to transplantation, the pulmonary vascular resistance in wood units [52], New York Heart Association (NYHA) classification, left ventricular ejection fraction (LVEF) and peak oxygen consumption (VO2) [53]. For the transplant operating procedure, we collect cross clamp -, bypass -, and re-perfusion times and the immediate post-transplant ECG rhythm.

The heart transplant follow-up data includes the date and cause of graft failure* if present (appendix table 3), any rejection episode* classified according to the 1990 ISHLT standardized cardiac biopsy grading of acute cellular rejection [54], and whether the rejection was clinical or subclinical*. Treatment of rejection episodes* is recorded together with the (combination of) drugs used. The recording of cardiac allograft diseases other than acute rejection is provided in table 3 of the appendix. Moreover, relevant transplant-related surgical complications are registered. We record early allograft LV dysfunction* occurring within the first days after transplantation and whether a VAD* was used or not. Regular functional cardiac monitoring that is performed during STCS cohort visits involves NYHA class*, VO2*, LVEF* (including diastolic or systolic dysfunction), and the current ECG rhythm*.

*Liver*

The comprehensive list of end-stage liver diseases recorded at baseline have been pre-specified (appendix table 1). We record the time point of disease diagnosis in the past, if possible, whether the disease was histologically proven and for hepatitis cases, the course of the disease (fulminant, acute, or chronic [55;56]). At baseline, we collect moreover the MELD - [57], and Child [58] scores, the state of encephalopathy and the presence of hepatorenal syndrome requiring dialysis or not. For primary liver cancer we collect comprehensive staging information. Namely the number of liver tumours overall and the number of tumours > 3 cm [59] based on the imaging modalities ultrasound, CT or MRI. The same information is additionally collected and validated based on the post-explantation pathological diagnosis. Based on the pathology reports, we also record the presence of angioinvasion [59]. For all liver tumours we record information on prior radiofrequency ablation (RFTA) or chemoembolization (TACE). In patients with hepatocellular carcinoma (HCC) we perform the tumour staging according to the Milan criteria [60]. During history taking we elicit the alcohol consumption behaviour as whether the patient “consumed alcohol at least once a week” in the past 6 months and if yes, we estimate the average daily consumption based on the type of alcohol consumed [61]. Liver-specific baseline physical assessment involves icterus, peripheral edema, encephalopathy, spider angioma, muscle waisting, and presence of ascites.

In liver transplant recipients, we collect an extended range of lab values, namely albumin in gramm/l, alpha-foetoprotein (AFP in μg/l), serum aminotransferases (ALAT, IU/l), total bilirubin (μmmol/l), factor V (in %), fibrinogen (gramm/l), INR and sodium (mmol/l). As time zero graft parameters we collect graft steatosis (in %), weight of the liver allograft, the type of transplantation performed (whole liver, split left or right graft, domino graft, reduced liver), and surgery duration.

After liver transplantation, we record graft failure with the date and the corresponding cause* if applicable (table 3). Acute cellular rejections (ACR) are recorded with the date of the episode*. We grade ACR using the rejection activity index (RAI)* [62]. Treatment of rejection episodes* is recorded together with the (combination of) drugs used. The occurrence of liver allograft diseases together with the date of disease diagnosis is recorded and provided in appendix table 3. Transplant-related complications are recorded as arterial thrombosis, portal vein thrombosis, biliary leak, biliary stenosis, bleeding, abdominal abscess, bowel perforation, surgical site-infection together with a number of interventions, if applicable, to treat complications or allograft diseases (table 4). We monitor transplant function by early allograft dysfunction/delayed graft function (DGF)* and its duration in days*. Due to the lack of a generally accepted definition of DGF, the diagnosis is at the treating physician’s discretion. From post-transplant biopsy specimens, we record the level of steatosis in percentage*, the level of fibrosis in stages F1 to F4* and the presence or absence of cirrhosis all based on histological diagnosis from biopsies*. In 2012, results of the Fibroscan as median and interquartile range in kPa together with the per cent success rate were added to the STCS database [63;64]. All baseline serology and lab parameters as well as the questions on alcohol consumption are longitudinally repeated in line with the baseline assessments at each regular cohort visit.

*Lung*

The comprehensive list of end-stage lung diseases ending in transplantation and STCS enrolment is provided in appendix table 2. We record the time point of disease diagnosis in the past, if possible. At baseline, we collect, the current and best FEV1*, the 6-minutes walking distance [65] and the NYHA class* with the dates corresponding to these assessments (most recent values prior to transplantation). Parameters of the actual transplantation involve the type of transplantation (left lung, right lung, double lung) and ischemia time.

During lung transplant follow-up, we record graft failure with the date of occurrence and the corresponding cause of failure* (appendix table 3). We collect all transbronchial biopsies, the presence of rejection*, if applicable the histologic ISHLT grading (A – D) of rejection severity [66] and the type of treatment used to treat rejection*. Allograft diseases include the presence of bronchiolitis obliterans syndrome (BOS) at each follow-up visits toghether with the BOS-grading system [67]. Further allograft diseases are specified in appendix table 3. Transplant-related complications involve bronchial complication, dehiscence, bronchial complication, stenosis, arterial complication, venous complication, transplantation-related re-surgery, surgical site-infection, or other complications. We determine the initial lung transplant function by measurement of total intubation time*, primary graft dysfunction (PGD)* defined and graded from 0 to 4 [68] and the need and duration of ECMO*. The duration of PGD is indicated in days. Further long-term lung functional parameters involve the determination of FEV1 current* (the measurement obtained during a cohort follow-up visit or the latest measurement pertaining to a cohort follow-up if a measurement occasion was missed) and FEV1 best*. The latter corresponds to the average of the two highest (not necessarily consecutive) measurements obtained at least 3 weeks apart and beyond three months after lung transplantation. FEV1 best measurements are made without preceding use of inhaled bronchodilator. At each follow-up visit, we moreover collect the results of the 6-minute walking test, BOS-grading* and the actual NYHA classification*.

*Pancreas and Islets*

The list of diseases leading to pancreas or islets transplantation and STCS enrolment is provided in appendix table 2. We record the time point of disease diagnosis in the past, if possible. At baseline, we collect the average daily insulin requirement during the previous 7 days in units/d and the latest proteinuria reading defined as the protein to creatinine ratio in mg/mmol.

Next to general organ-related data, we determine an extended range of routine lab values at baseline and at each follow-up visit. The C-peptide and insulinemia are determined as basal and as stimulated values in picomol/l and in mmol/l, respectively. As stimulation methods, we use glucagon, arginin, intravenous glucose, oral glucose or the mixed meal methods. We moreover measure fasting glucose in mmol/l, fasting total cholesterol, triglycerides, HbA1c and the MAGE score in mmol/l [69]. Related to the pancreas transplantation procedure, we collect the cold ischemia time, for islets the number of islet equivalents infused, the access for transplantation (percutaneous or by open surgery), and the transplantation site (intrahepatic or other sites).

For combined kidney-pancreas double transplantations, the STCS patient-case system links the two organs to one case but flexibly follows each organ separately. During the pancreas post-transplant course, we observe the time point of graft failure and its cause* (appendix table 3). Rejection episodes* are recorded as either biopsy-proven or clinically suspected with the type of treatment used to treat rejection*. Complications are recorded as arterial thrombosis, portal vein thrombosis, bleeding, peritonitis, pancreatitis, abdominal abscess, bowel perforation or surgical site-infection in pancreas and islet transplantation. In pancreas and islet transplantation, we additionally collect post-transplant procedure-specific interventions. Regular post-transplant lab assessments follow at each cohort visit in line with the baseline assessments. Transplant function involves early allograft dysfunction (DGF)* and the duration of DGF*. Due to the lack of a generally accepted definition of DGF, the diagnosis is at the treating physician’s discretion. In line with the baseline assessment, we collect the average daily insulin requirement during the previous 7 days* and proteinuria (protein-creatinine ratio)* at each scheduled cohort visit. As summary interpretation, we classify the glycemic control during each follow-up visit as normal, impaired glucose tolerance and presence of diabetes* [6].

*Small bowel*

The comprehensive list of conditions leading to small bowel transplantation has been pre-specified (appendix table 2). We record the time point of disease diagnosis in the past, if possible, and whether the disease was histologically proven. At baseline, we moreover ask for the presence of complete or incomplete malabsorption, the length of the remaining bowel in the case of short bowel syndrome, whether the ileo-caecal valve was preserved, and the portion of the preserved colon following bowel resection (right colon, transverse colon, descending colon, sigmoid colon, rectum). Finally, we collect the duration of total parenteral nutrition and complications thereof (thrombosis of central venous access, septic shock, line sepsis with resistant bacteria, systemic fungal infection, endocarditis, brain abscess, other septic complications, fatty liver degeneration/steatosis, reversible fibrosis, irreversible fibrosis, other).

At baseline, we collect an extended range of lab values in small bowel recipients namely albumin in gram/l, ALAT and ASAT (IU/l), total and conjugated bilirubin (μmmol/l), citrulin, factor V (in %), fibrinogen (gram/l), prealbumin (mg/l), INR and prothrombine time (PT), fasting triglycerides (mmol/l) and maximal D-xylose absorption in mmol/l. As baseline graft parameters, we collect the type of transplantation performed (whole small bowel, reduced small bowel, combined liver-small bowel, multivisceral), and the length of the implanted graft.

During the post-transplant course, we observe time to diagnosis of allograft diseases and/or graft failure with the corresponding cause* (appendix table 3). Rejection episodes* are recorded as acute cellular rejection (ACR) with the severity mild, intermediate, moderate, severe, or clinically suspected rejection that are not biopsy proven. The type of treatment used to treat rejection* is collected if applicable. Complications are recorded with the date of occurrence and the corresponding type of complication (arterial thrombosis, venous thrombosis, peritonitis, bleeding, abdominal abscess, bowel perforation, and surgical site-infection) together with post-transplant procedure-specific interventions. Regular post-transplant lab assessments follow at each cohort visit in line with the baseline assessments as specified above. Transplant function involves early allograft dysfunction (DGF)* with the duration of DGF* and the need for partial or total parenteral nutrition*. Due to the lack of a generally accepted definition of DGF, the diagnosis is at the treating physician’s discretion.

**Bio sampling**

After a review of recent literature to look for which sample type and which schedule of sampling could cover testing needs other than routine blood counts and chemistry, we came to a proposal of sampling representing a compromise between testing needs and resources necessary to harvest and store such samples.

Three types of samples have been retained: (i) genomic DNA extracted from peripheral blood mononuclear cells harvested at the time of transplantation for genetic studies (ii) plasma for antibody and other blood solutes testing. Plasma was preferred to serum as this sample could be produced as a by-product of peripheral blood mononuclear cell sample production, and as many current antibody tests are validated using either sample type. (iii) Live peripheral blood mononuclear cells (PBMC) for functional analysis, including immune responses.

Samples sets are harvested at the following time points: time of transplantation (T0), i.e when the recipient arrives at the hospital after being called for transplantation. For live donor transplantation, the samples can be harvested a maximum 15 days before the scheduled transplantation day. Further time points are 6 and 12 months (T6 and T12) after transplantation (samples have to be taken within a time window of +15 days around the time point).

The samples are processed and stored locally. Genomic DNA is obtained using QIAamp DNA Blood kit or Genoprep, Qiagen, or Maxwell purification kit, Promega, according to the manufacturer instruction. Two 100 μl aliquots containing about 10 μg DNA are prepared from about 2ml whole blood. DNA concentration and purity is measured by spectrophotometry (Concentration range: 50-150 ng/microliter, Purity: OD260/280: 1.8-2), and average fragment molecular weight by pulse field gel electrophoresis on selected samples. These two storage aliquots are kept in polypropylene screwcap rubber O-ring tubes and stored at –20°C. When DNA is requested for studies, a tenfold working dilution (10 ng/μL) is made out of the storage aliquot. For standard PCR studies, 10 μL, i.e. 100 ng DNA are sent out. The working dilution tube is kept at 4°C. In addition, when the first DNA storage aliquot is exhausted, a 10-100 ng sample is used for a genomic amplification procedure so that a second storage aliquot of DNA is restored. The amplification is performed using a kit (e.g. Repli-g, Qiagen, Genomiphi, Amersham) according to the manufacturer instruction. The blood sample for DNA preparation is harvested only once, at baseline, or subsequently if not available at that time.

At baseline, 6 month and 12 month, a 40-45 ml EDTA blood sample is taken for plasma and PBMC preparation. Plasma is obtained by an initial centrifugation step and aliquotted into 5x2 ml in polypropylene screwcap rubber O-ring tubes, and stored at –80°C. PBMC are then separated by centrifugation on a Ficoll-Hypaque layer , washed, counted and aliquotted in 5x1 ml freeze medium at a concentration of 8-15 millions cells/ml in cryotubes frozen using a controlled freezing procedure and kept in liquid nitrogen. Media, including serum used are checked endotoxin-free and non mitogenic. Serum is heat-inactivated. The yield, viability and stimulation of cells is periodically checked using volunteer blood.

Samples are registered both in the local laboratory information system and in the central cohort database. The STCS lab group consists of one center representative each and addresses all issues in this respect. This group has produced written procedures to ensure consistency in samples processing and storage (live PBMCs and plasma, and DNA). In addition, the group has organized quality testing using volunteer blood, to check DNA purity and average fragment size, and live cells viability, and absence of mitogenic activity in the PBMC separation procedure. Finally, the lab group chair collects information from investigators as to the quality of samples used in ongoing studies.

**Other data definitions**

In the case of missing data, each database field requires a precise definition of the missing data generating process. We therefore distinguish between values that are “not applicable” (e.g. an event date when no event occurred), “not done” (e.g. the local site did not request a lab assessment), “unknown” (information cannot be obtained, neither via the patient nor the hospital), or “refused” (the patient actively refused answers or blood sampling). Only missing data that cannot be resolved and that do not fall into one of these defined categories will be queried by the STCS data-centre and site staff.

**Appendix Tables and Figures**

**Table 1**: STCS immediate and underlying causes of death [12]

| **Immediate causes of death** |
| --- |
| Graft dysfunction |
| Surgery-related |
| Hemodynamic failure |
| Infectious disease |
| Multi-organ failure |
| Pulmonary embolism (PE) |
| Suicide |
| Acute respiratory distress syndrome / alveolar damage (ARDS) |
| Chronic obstructive pulmonary disease / Asthma |
| Liver failure |
| Digestive haemorrhage |
| Cerebrovascular diseases (Stroke ischemic or hemorrage) |
| Coronary heart disease |
| Cardiac failure / right heart failure |
| Sudden death |
| Dementia, M. Parkinson, degenerative CNS diseases |
| Aortic aneurysm rupture |
| Acute pancreatitis |
| Pulmonary hemorrhage |
| Acute renal failure |
| Hypoxic-ischemic brain injury |
| Coagulopathy |
| Calciphylaxis / Vasculitis |
| Bowel ischemia / infarction |
| Brain cancer |
| Breast cancer |
| Cervix - Uterus - Adnex |
| Colon cancer |
| Kaposi's sarkoma |
| Kidney cancer |
| Leukemia |
| Myeloproliferative disorder / myelodysplastic syndrome |
| Liver cancer |
| Lung cancer |
| Lymphoma |
| Prostate cancer |
| Skin, squamous cell carcinoma |
| Skin, basal cell carcinoma |
| Skin, melanoma |
| Urothel / bladder cancer |
| Neuroendocrine tumor |
| Thyroid cancer |
| Testicular cancer |
| Multiple myeloma / Amyloidosis |
| Sarkoma |
| Cancer of unknown primary origin |
| GvHD |
| Trauma |
| Gangrene |
| Unobserved death |
| Other |
| Unknown |

| **Underlying cause leading to death** |
| --- |
| Pulmonary artery hypertension |
| Diabetes mellitus Typ 1 |
| Diabetes mellitus Typ 2 |
| Peripheral vascular disease |
| Chronic obstructive pulmonary disease / Asthma |
| Cerebrovascular diseases (Stroke ischemic or hemorragic) |
| Coronary heart disease |
| Dementia, M. Parkinson, degenerative CNS diseases |
| Acute renal failure |
| Calciphylaxis / vasculitis |
| Brain cancer |
| Breast cancer |
| Cervix - Uterus - Adnex |
| Colon cancer |
| Kaposi's sarkoma |
| Kidney cancer |
| Leukaemia |
| Myeloproliferative disorder / myelodysplastic syndrome |
| Liver cancer |
| Lung cancer |
| Lymphoma |
| Prostate cancer |
| Skin, squamous cell carcinoma |
| Skin, basal cell carcinoma |
| Skin, melanoma |
| Urothel / bladder cancer |
| Neuroendocrine tumor |
| Thyroid cancer |
| Testicular cancer |
| Multiple myeloma / amyloidosis |
| Sarkoma |
| Cancer of unknown primary origin |
| Graft versus Host Disease (GvHD) |
| Other |
| Unknown |

**Table 2:** Pre-specified native diseases leading to end-stage organ failure and transplantation as defined in the STCS.

| **Organ** | **Native diseases leading to transplantation** |
| --- | --- |
| Heart | Ischemic heart disease  Dilated cardiomyopathy  Hypertrophic cardiomyopathy  Valvular heart disease  Congenital heart disease  Arrhythmogenic right ventricular dysplasia (ARVD)  Other arrhythmogenic heart disease  Restrictive cardiomyopathy  Uhl’s disease  Previous allograft failure  Cause unknown  Other causes |
| Kidney | Diabetic nephropathy  Hypertensive/renovascular nephrosclerosis  Glomerulonephritis/vasculitis  Polycystic kidney disease  Hereditary kidney disease other than polycystic kidney disease  Interstitial nephritis, not hereditary  HIV nephropathy  Obstructive nephropathy/reflux/pyelonephritis  Previous allograft failure  Congenital disease/malformation  Cause unknown  Other causes |
| Liver | Viral  Hepatitis B  Hepatitis B-D  Hepatitis C  Toxic  Drug-induced  Alcoholic liver disease  Mushroom poisoning  Cholostatic disease  Primary biliary cirrhosis (PBC)  Secondary biliary cirrhosis  Sclerosing cholangitis  Progressive Familial Intrahepatic Cholostasis (PFIC)  Cystic Fibrosis (CF)  Extrahepatic biliary atresia (congenital biliary)  Cancer  Hepatocellular carcinoma (HCC)  Cholangiocarcinoma  Epitheloid hemangioendothelioma  Metabolic  Wilson disease  Alfa-1 antitrypsin deficiency  Crigler-Najar syndrome  Hemochromatosis  Glycogenosis  Oxalosis  Other  Budd-Chiari syndrome  Benign liver tumors  Echinococcosis  Cryptogenic/idiopathic liver disease  Autoimmune hepatitis  Previous allograft failure  Cause unknown  Other causes |
| Lung | Chronic obstructive pulmonary disease (COPD) or emphysema  Idiopathic Pulmonary Fibrosis (IPF)  Interstitial Lung Disease (all others except IPF) (ILD)  Cystic Fibrosis (CF)  Alpha 1 Anti-Trypsin deficiency (AAT)  Pulmonary Arterial Hypertension (PAH)  Bronchiectasis (BCT)  Congenital Heart Disease (CHD)  Lymphangioleiomyomatosis (LAM)  Sarcoidosis (SAR)  Previous allograft failure  Cause unknown  Other causes |
| Pancreas / islets | Diabetes mellitus type 1  Diabetes mellitus type 2  Postpancreatectomy diabetes  Cystic fibrosis (CF)  Previous allograft failure  Cause unknown  Other causes |
| Small bowel | Short bowel syndrome  Mesenteric thrombosis, intestinal infarction  Intestinal volvulus  Crohn's disease  Intestinal atresia  Necrotizing enterocolitis  Gastroschisis  Intestinal/mesenteric trauma  Motility disorder and malabsorption  Hirschsprung's disease  Aganglionosis  Diabetic enteropathy  Intestinal malabsorption disorder  Benign tumors  Gardner's syndrome  Desmoid tumor  Previous allograft failure  Cause unknown  Other causes |

**Table 3:** Transplant-specific outcome events and diseases as defined in the STCS.

| **Organ** | **Graft failure** | **Allograft disease*** |
| --- | --- | --- |
| Kidney | Immunological  Renal artery thrombosis  Renal vein thrombosis  disease  Allograft disease *  Technical / complication **  Other causes  Cause unknown | Acute tubular necrosis (ATN)  Thrombotic microangiopathy  BKV nephropathy (SV40+)  Chronic allograft nephropathy (CAN)  Calcineurin inhibitor (CNI ) toxicity  Diabetic nephropathy  Other  Recurrence of native kidney disease |
| Heart | Immunological  Right heart failure  Acute ischemic  Chronic ischemic  Hemodynamic acute graft failure  Allograft disease *  Technical / complication **  Other causes  Cause unknown | Transplant vasculopathy (CAV)  Arrhythmia with device implantation (pacemaker or ICD)  Arrhythmia without device implantation (pacemaker or ICD)  Transplant valvulopathy  Infection other than surgical site  CNI-induced toxicity  Recurrence of native cardiac disease |
| Liver | Hyperacute rejection  Arterial thrombosis  Fulminant hepatitis  Allograft disease *  Technical / complication **  Other causes  Cause unknown | Hepatitis B (de novo)  Hepatitis B-D (de novo)  Hepatitis C (de novo)  Hepatocellular carcinoma (HCC) (de novo)  Steato-hepatitis (de novo)  Chronic rejection [62]  Recurrence of native liver disease |
| Lung | Acute immunological  Allograft disease*  Technical / complication **  Other causes  Cause unknown | Bronchiolitis obliterans Syndrome (BOS) [70]  Tumor  Infection  Pulmonary artery hypertension  Restrictive allograft syndrome (RAS)  Recurrence of native lung disease |
| Pancreas | Primary non-function  Acute rejection (Immunological)  Recurrence of autoimmunity i.e. recurrence of type 1 diabetes  Arterial thrombosis  Portal vein thrombosis  Pancreatitis  Graft exhaustion/chronic rejection  Technical / complication **  Other causes  Cause unknown |  |
| Islets | Acute rejection (Immunological)  Technical graft loss (early non-immunological)  Recurrence of autoimmunity i.e. recurrence of type 1 diabetes  Arterial thrombosis  Portal vein thrombosis  Graft exhaustion/chronic rejection  Technical / complication **  Other causes  Cause unknown |  |
| Small bowel | Immunological, rejection  Septic  Arterial thrombosis  Venous allograft thrombosis  Allograft disease*  Technical / complication **  Other causes  Cause unknown | Infectious enteritis  Chronic rejection  Recurrence of the native small bowel disease |

* Allograft diseases are recorded during follow-up and are consistently part of the causes of allograft failure.

** Appendix Table 4

**Table 4:** Transplant-related complications and interventions as defined in the STCS

| **Organ** | **Transplant-related complications** | **Transplant-related interventions** |
| --- | --- | --- |
| Kidney** | Urine leak  Lymphocele  Obstruction  Renal artery stenosis/Kincking  Renal artery thrombosis  Renal vein thrombosis  Transplantation-related re-surgery  Surgical site-infection  Intraabdominal infection  Biopsy-related complication  Other  Unknown | Not defined |
| Heart | Lymphocele  Hemorrhagic complication  Diaphragma paralysis  Transplantation-related re-surgery  Surgical site-infection  Air embolism  Biopsy-related complication  Other  Unknown | Not defined |
| Liver | Arterial thrombosis  Portal vein thrombosis  Biliary leak  Bililary stenosis  Bleeding  Abdominal abcess  Bowel perforation  Surgical site-infection  Cholangitis  Biopsy-related complication  Other  Unknown | Surgical artery reconstruction  Arterial stent placement  Arterial balloon dilatation  Arterial angioplasty  Surgical portal vein reconstruction  Portal vein stent placement  Portal vein angioplasty  Surgical biliary reconstruction  Surgery of biliary stenosis  Biliary stent placement  Biliary balloon dilatation  Laparotom resection  ERCP  Gastroscopy  Colonoscopy  Other  Unknown |
| Lung | Bronchial complication, dehiscence  Bronchial complication, stenosis  Arterial complication  Venous complication  Transplantation-related re-surgery  Surgical site-infection  Aspiration  Biopsy-related complication  Diaphragmatic dysfunction  Other  Unknown | Not defined |
| Pancreas | Arterial thrombosis  Portal vein thrombosis  Bleeding  Peritonitis  Pancreatitis  Abdominal abscess  Bowel perforation  Surgical site-infection  Biopsy-related complication  Other  Unknown | Arterial reconstruction  Thrombectomy  Pancreatectomy  Abdominal wash out  Haemostasis  Drainage  Other  Unknown |
| Islets | Arterial thrombosis  Portal vein thrombosis  Bleeding  Peritonitis  Pancreatitis  Abdominal abscess  Bowel perforation  Surgical site-infection  Other  Unknown | Arterial reconstruction  Thrombectomy  Abdominal wash out  Hemostasis  Drainage  Other  Unknown |
| Small bowel | Arterial thrombosis  Venous allograft thrombosis  Peritonitis  Bleeding  Abdominal abscess  Bowel perforation  Surgical site-infection  Biopsy-related complication  Other  Unknown | Arterial reconstruction  Venous reconstruction  Exploratory laparotomy  Abdominal wash out  Explantation  Other  Unknown |

Reference List

1. Thygesen K, Alpert JS, White HD. Universal definition of myocardial infarction. Eur Heart J 2007 Oct;28(20):2525-38.

2. Adams HP, Jr., del ZG, Alberts MJ, Bhatt DL, Brass L, Furlan A, et al. Guidelines for the early management of adults with ischemic stroke: a guideline from the American Heart Association/American Stroke Association Stroke Council, Clinical Cardiology Council, Cardiovascular Radiology and Intervention Council, and the Atherosclerotic Peripheral Vascular Disease and Quality of Care Outcomes in Research Interdisciplinary Working Groups: the American Academy of Neurology affirms the value of this guideline as an educational tool for neurologists. Stroke 2007 May;38(5):1655-711.

3. Broderick J, Connolly S, Feldmann E, Hanley D, Kase C, Krieger D, et al. Guidelines for the management of spontaneous intracerebral hemorrhage in adults: 2007 update: a guideline from the American Heart Association/American Stroke Association Stroke Council, High Blood Pressure Research Council, and the Quality of Care and Outcomes in Research Interdisciplinary Working Group. Stroke 2007 Jun;38(6):2001-23.

4. Norgren L, Hiatt WR, Dormandy JA, Nehler MR, Harris KA, Fowkes FG. Inter-Society Consensus for the Management of Peripheral Arterial Disease (TASC II). J Vasc Surg 2007 Jan;45 Suppl S:S5-67.

5. Tutone VK, Mark PB, Stewart GA, Tan CC, Rodger RS, Geddes CC, et al. Hypertension, antihypertensive agents and outcomes following renal transplantation. Clin Transplant 2005 Apr;19(2):181-92.

6. Alberti KG, Zimmet PZ. Definition, diagnosis and classification of diabetes mellitus and its complications. Part 1: diagnosis and classification of diabetes mellitus provisional report of a WHO consultation. Diabet Med 1998 Jul;15(7):539-53.

7. Third Report of the National Cholesterol Education Program (NCEP) Expert Panel on Detection, Evaluation, and Treatment of High Blood Cholesterol in Adults (Adult Treatment Panel III) final report. Circulation 2002 Dec 17;106(25):3143-421.

8. Freifeld AG, Bow EJ, Sepkowitz KA, Boeckh MJ, Ito JI, Mullen CA, et al. Clinical practice guideline for the use of antimicrobial agents in neutropenic patients with cancer: 2010 update by the infectious diseases society of america. Clin Infect Dis 2011 Feb 15;52(4):e56-e93.

9. O'Brien E, Asmar R, Beilin L, Imai Y, Mancia G, Mengden T, et al. Practice guidelines of the European Society of Hypertension for clinic, ambulatory and self blood pressure measurement. J Hypertens 2005 Apr;23(4):697-701.

10. World Health Organisation (2007). International statistical classification of diseases and related health problems (ICD-10), 10th Revision. 2010. 11-8-2010.

11. Centres for Disease Control and Prevention. 2012. 15-2-2012.

12. Sanroman BB, Vazquez ME, Pertega DS, Veiga BA, Carro RE, Mosquera RJ. Autopsy-determined causes of death in solid organ transplant recipients. Transplant Proc 2004 Apr;36(3):787-9.

13. The immunocompromised Host Society. Guidelines for the Management of Bacterial, Fungal and Viral Infections. 2012. 12-1-2012.

14. IDSA Practice Guidelines. 2012. 12-1-2012.

15. Jones E. Translation of quantitative measures for use in cross-cultural research. Nurs Res 1987 Sep;36(5):324-7.

16. Schweizerische Eidgenossenschaft, Swiss Health Observatory (OBSAN). 2010. 11-8-2010.

17. Schweizerische Eidgenossenschaft, Schweizerische Gesundheitsbefragung 2007. 2010. 11-8-2010.

18. Sendi P, Schellenberg F, Ungsedhapand C, Kaufmann GR, Bucher HC, Weber R, et al. Productivity costs and determinants of productivity in HIV-infected patients. Clin Ther 2004 May;26(5):791-800.

19. Glass TR, De GS, Hirschel B, Battegay M, Furrer H, Covassini M, et al. Self-reported non-adherence to antiretroviral therapy repeatedly assessed by two questions predicts treatment failure in virologically suppressed patients. Antivir Ther 2008;13(1):77-85.

20. Glass TR, De GS, Weber R, Vernazza PL, Rickenbach M, Furrer H, et al. Correlates of self-reported nonadherence to antiretroviral therapy in HIV-infected patients: the Swiss HIV Cohort Study. J Acquir Immune Defic Syndr 2006 Mar;41(3):385-92.

21. De GS, Denhaerynck K, Schafer-Keller P, Bock A, Steiger J. Supporting medication adherence in renal transplantation--the SMART study. Swiss Med Wkly 2007 Mar 2;137 Suppl 155:125S-7S.

22. Deschamps AE, De GS, Vandamme AM, Bobbaers H, Peetermans WE, Van WE. Diagnostic value of different adherence measures using electronic monitoring and virologic failure as reference standards. AIDS Patient Care STDS 2008 Sep;22(9):735-43.

23. Elzi L, Spoerl D, Voggensperger J, Nicca D, Simcock M, Bucher HC, et al. A smoking cessation programme in HIV-infected individuals: a pilot study. Antivir Ther 2006;11(6):787-95.

24. Glanz K, Yaroch AL, Dancel M, Saraiya M, Crane LA, Buller DB, et al. Measures of sun exposure and sun protection practices for behavioral and epidemiologic research. Arch Dermatol 2008 Feb;144(2):217-22.

25. World Health Organisation (2007). International statistical classification of diseases and related health problems (ICD-10), 10th Revision. 2010. 11-8-2010.

26. Zipfel S, Schneider A, Wild B, Lowe B, Junger J, Haass M, et al. Effect of depressive symptoms on survival after heart transplantation. Psychosom Med 2002 Sep;64(5):740-7.

27. Herrmann C. International experiences with the Hospital Anxiety and Depression Scale--a review of validation data and clinical results. J Psychosom Res 1997 Jan;42(1):17-41.

28. Bjelland I, Dahl AA, Haug TT, Neckelmann D. The validity of the Hospital Anxiety and Depression Scale. An updated literature review. J Psychosom Res 2002 Feb;52(2):69-77.

29. Stafford L, Berk M, Jackson HJ. Validity of the Hospital Anxiety and Depression Scale and Patient Health Questionnaire-9 to screen for depression in patients with coronary artery disease. Gen Hosp Psychiatry 2007 Sep;29(5):417-24.

30. Goetzmann L, Klaghofer R, Wagner-Huber R, Halter J, Boehler A, Muellhaupt B, et al. Quality of life and psychosocial situation before and after a lung, liver or an allogeneic bone marrow transplant. Swiss Med Wkly 2006 Apr 29;136(17-18):281-90.

31. Estraviz B, Quintana JM, Valdivieso A, Bilbao A, Ortiz de UJ, Sarabia S. [Psychometric properties of a quality of life questionnaire specific to liver transplant]. Rev Esp Enferm Dig 2007 Jan;99(1):13-8.

32. Smeritschnig B, Jaksch P, Kocher A, Seebacher G, Aigner C, Mazhar S, et al. Quality of life after lung transplantation: a cross-sectional study. J Heart Lung Transplant 2005 Apr;24(4):474-80.

33. Noohi S, Khaghani-Zadeh M, Javadipour M, Assari S, Najafi M, Ebrahiminia M, et al. Anxiety and depression are correlated with higher morbidity after kidney transplantation. Transplant Proc 2007 May;39(4):1074-8.

34. Achermann P. The two-process model of sleep regulation revisited. Aviat Space Environ Med 2004 Mar;75(3 Suppl):A37-A43.

35. Borbely AA. A two process model of sleep regulation. Hum Neurobiol 1982;1(3):195-204.

36. Daan S, Beersma DG, Borbely AA. Timing of human sleep: recovery process gated by a circadian pacemaker. Am J Physiol 1984 Feb;246(2 Pt 2):R161-R183.

37. RAND. Kidney Disease and Quality of Life™ Short Form (KDQOL-SF™). 2010. 12-8-2010.

38. Gorodetskaya I, Zenios S, McCulloch CE, Bostrom A, Hsu CY, Bindman AB, et al. Health-related quality of life and estimates of utility in chronic kidney disease. Kidney Int 2005 Dec;68(6):2801-8.

39. Elder SJ, Pisoni RL, Akizawa T, Fissell R, Andreucci VE, Fukuhara S, et al. Sleep quality predicts quality of life and mortality risk in haemodialysis patients: results from the Dialysis Outcomes and Practice Patterns Study (DOPPS). Nephrol Dial Transplant 2008 Mar;23(3):998-1004.

40. Burkhalter H, Sereika SM, Engberg S, Wirz-Justice A, Steiger J, De GS. Validity of 2 sleep quality items to be used in a large cohort study of kidney transplant recipients. Prog Transplant 2011 Mar;21(1):27-35.

41. Almenar-Pertejo M, Almenar L, Martinez-Dolz L, Campos J, Galan J, Girones P, et al. Study on health-related quality of life in patients with advanced heart failure before and after transplantation. Transplant Proc 2006 Oct;38(8):2524-6.

42. McLernon DJ, Dillon J, Donnan PT. Health-state utilities in liver disease: a systematic review. Med Decis Making 2008 Jul;28(4):582-92.

43. Russell RT, Feurer ID, Wisawatapnimit P, Pinson CW. The validity of EQ-5D US preference weights in liver transplant candidates and recipients. Liver Transpl 2009 Jan;15(1):88-95.

44. Saeed I, Rogers C, Murday A. Health-related quality of life after cardiac transplantation: results of a UK National Survey with Norm-based Comparisons. J Heart Lung Transplant 2008 Jun;27(6):675-81.

45. Santana MJ, Feeny D, Johnson JA, McAlister FA, Kim D, Weinkauf J, et al. Assessing the use of health-related quality of life measures in the routine clinical care of lung-transplant patients. Qual Life Res 2010 Apr;19(3):371-9.

46. Bryan S, Longworth L. Measuring health-related utility: why the disparity between EQ-5D and SF-6D? Eur J Health Econ 2005 Sep;6(3):253-60.

47. Longworth L, Bryan S. An empirical comparison of EQ-5D and SF-6D in liver transplant patients. Health Econ 2003 Dec;12(12):1061-7.

48. Swiss National Foundation for organ donation and transplantation. 2010. 13-2-2012.

49. Solez K, Colvin RB, Racusen LC, Sis B, Halloran PF, Birk PE, et al. Banff '05 Meeting Report: differential diagnosis of chronic allograft injury and elimination of chronic allograft nephropathy ('CAN'). Am J Transplant 2007 Mar;7(3):518-26.

50. Solez K, Colvin RB, Racusen LC, Haas M, Sis B, Mengel M, et al. Banff 07 classification of renal allograft pathology: updates and future directions. Am J Transplant 2008 Apr;8(4):753-60.

51. Montgomery RA, Hardy MA, Jordan SC, Racusen LC, Ratner LE, Tyan DB, et al. Consensus opinion from the antibody working group on the diagnosis, reporting, and risk assessment for antibody-mediated rejection and desensitization protocols. Transplantation 2004 Jul 27;78(2):181-5.

52. Baim D. Grossman's Cardiac Catheterization, Angiography, and Intervention. 7th Ed. ed. Lipincott Williams & Wilkins; 2006.

53. Gibbons RJ, Balady GJ, Bricker JT, Chaitman BR, Fletcher GF, Froelicher VF, et al. ACC/AHA 2002 guideline update for exercise testing: summary article: a report of the American College of Cardiology/American Heart Association Task Force on Practice Guidelines (Committee to Update the 1997 Exercise Testing Guidelines). Circulation 2002 Oct 1;106(14):1883-92.

54. Billingham ME, Cary NR, Hammond ME, Kemnitz J, Marboe C, McCallister HA, et al. A working formulation for the standardization of nomenclature in the diagnosis of heart and lung rejection: Heart Rejection Study Group. The International Society for Heart Transplantation. J Heart Transplant 1990 Nov;9(6):587-93.

55. Liaw YF, Leung N, Guan R, Lau GK, Merican I, McCaughan G, et al. Asian-Pacific consensus statement on the management of chronic hepatitis B: a 2005 update. Liver Int 2005 Jun;25(3):472-89.

56. Ghany MG, Strader DB, Thomas DL, Seeff LB. Diagnosis, management, and treatment of hepatitis C: an update. Hepatology 2009 Apr;49(4):1335-74.

57. Kamath PS, Wiesner RH, Malinchoc M, Kremers W, Therneau TM, Kosberg CL, et al. A model to predict survival in patients with end-stage liver disease. Hepatology 2001 Feb;33(2):464-70.

58. Pugh RN, Murray-Lyon IM, Dawson JL, Pietroni MC, Williams R. Transection of the oesophagus for bleeding oesophageal varices. Br J Surg 1973 Aug;60(8):646-9.

59. Vauthey JN, Lauwers GY, Esnaola NF, Do KA, Belghiti J, Mirza N, et al. Simplified staging for hepatocellular carcinoma. J Clin Oncol 2002 Mar 15;20(6):1527-36.

60. Mazzaferro V, Regalia E, Doci R, Andreola S, Pulvirenti A, Bozzetti F, et al. Liver transplantation for the treatment of small hepatocellular carcinomas in patients with cirrhosis. N Engl J Med 1996 Mar 14;334(11):693-9.

61. Conen A, Fehr J, Glass TR, Furrer H, Weber R, Vernazza P, et al. Self-reported alcohol consumption and its association with adherence and outcome of antiretroviral therapy in the Swiss HIV Cohort Study. Antivir Ther 2009;14(3):349-57.

62. Demetris A, Adams D, Bellamy C, Blakolmer K, Clouston A, Dhillon AP, et al. Update of the International Banff Schema for Liver Allograft Rejection: working recommendations for the histopathologic staging and reporting of chronic rejection. An International Panel. Hepatology 2000 Mar;31(3):792-9.

63. Sandrin L, Fourquet B, Hasquenoph JM, Yon S, Fournier C, Mal F, et al. Transient elastography: a new noninvasive method for assessment of hepatic fibrosis. Ultrasound Med Biol 2003 Dec;29(12):1705-13.

64. Kettaneh A, Marcellin P, Douvin C, Poupon R, Ziol M, Beaugrand M, et al. Features associated with success rate and performance of FibroScan measurements for the diagnosis of cirrhosis in HCV patients: a prospective study of 935 patients. J Hepatol 2007 Apr;46(4):628-34.

65. Enright PL, McBurnie MA, Bittner V, Tracy RP, McNamara R, Arnold A, et al. The 6-min walk test: a quick measure of functional status in elderly adults. Chest 2003 Feb;123(2):387-98.

66. Stewart S, Fishbein MC, Snell GI, Berry GJ, Boehler A, Burke MM, et al. Revision of the 1996 working formulation for the standardization of nomenclature in the diagnosis of lung rejection. J Heart Lung Transplant 2007 Dec;26(12):1229-42.

67. Boehler A, Estenne M. Post-transplant bronchiolitis obliterans. Eur Respir J 2003 Dec;22(6):1007-18.

68. Christie JD, Carby M, Bag R, Corris P, Hertz M, Weill D. Report of the ISHLT Working Group on Primary Lung Graft Dysfunction part II: definition. A consensus statement of the International Society for Heart and Lung Transplantation. J Heart Lung Transplant 2005 Oct;24(10):1454-9.

69. Service FJ, Molnar GD, Rosevear JW, Ackerman E, Gatewood LC, Taylor WF. Mean amplitude of glycemic excursions, a measure of diabetic instability. Diabetes 1970 Sep;19(9):644-55.

70. Estenne M, Maurer JR, Boehler A, Egan JJ, Frost A, Hertz M, et al. Bronchiolitis obliterans syndrome 2001: an update of the diagnostic criteria. J Heart Lung Transplant 2002 Mar;21(3):297-310.
